# Opioid Substitution Treatment Planning in a Disaster Context: Perspectives from Emergency Management and Health Professionals in Aotearoa/New Zealand

**DOI:** 10.3390/ijerph13111122

**Published:** 2016-11-10

**Authors:** Denise Blake, Antonia Lyons

**Affiliations:** 1 Joint Centre for Disaster Research, School of Psychology, Massey University, Wellington 6140, New Zealand; 2 School of Psychology, Massey University, Wellington 6140, New Zealand; A.Lyons@massey.ac.nz

**Keywords:** opioid substitution treatment, OST, vulnerability, disaster, preparedness, planning

## Abstract

Opioid Substitution Treatment (OST) is a harm reduction strategy enabling opiate consumers to avoid withdrawal symptoms and maintain health and wellbeing. Some research shows that within a disaster context service disruptions and infrastructure damage affect OST services, including problems with accessibility, dosing, and scripts. Currently little is known about planning for OST in the reduction and response phases of a disaster. This study aimed to identify the views of three professional groups working in Aotearoa/New Zealand about OST provision following a disaster. In-depth, semi-structured interviews were conducted with 17 service workers, health professionals, and emergency managers in OST and disaster planning fields. Thematic analysis of transcripts identified three key themes, namely “health and wellbeing”, “developing an emergency management plan”, and “stock, dose verification, and scripts” which led to an overarching concept of “service continuity in OST preparedness planning”. Participants viewed service continuity as essential for reducing physical and psychological distress for OST clients, their families, and wider communities. Alcohol and drug and OST health professionals understood the specific needs of clients, while emergency managers discussed the need for sufficient preparedness planning to minimise harm. It is concluded that OST preparedness planning must be multidisciplinary, flexible, and inclusive.

## 1. Introduction

Providing access to opioid substitution treatment (OST) is crucial to the ongoing health and wellbeing of opioid users, their families, communities, and wider social systems. However, in an immediate post-disaster context, access to opioid interventions can be problematic [1,2,3], although research into OST disaster planning and response is scarce. Having a pre-existing mental and physical health condition, such as an opioid-related disorder, can produce increased vulnerability for an already marginalised group and poorer health and wellbeing outcomes during and immediately following a disaster [4,5,6,7]. A disaster amplifies social vulnerabilities, inequalities, and hardships. Often poverty, inadequate housing, chronic health conditions, and poor health care already exist, so essential resources that might enable resilient coping are not available [8,9]. Without resources such as cell phone credit, Internet access, or transport, organising OST is problematic. Understanding the specificities of particular groups enables inclusive preparedness [10], and assists in addressing the personal and public health effects of opioid dependence in a disaster context [4].

Opioid related disorders are characterized by a set of problematic behaviours, including taking the substance for longer periods of time than intended, being unable to cease drug use despite wanting to, and experiencing obsessive overpowering cravings for the substance. Continued drug use frequently occurs despite consequent problems at work, school, or within relationships. There are also behavioural risks, such as operating machinery or driving under the influence [11]. The social implications of drug using behaviour (e.g., familial relationships breakdowns and loss of productivity) can be distressing for the person, their family, and friends [12,13]. Drug dependency also impinges on wider social systems, such as medical care and essential social support services [14] due to the high social and health needs of the clients.

With prolonged opioid use, physical tolerance ensues and increased amounts are required to experience intoxication. Withdrawal symptoms include agitation, nausea, muscle aches, diarrhoea, and vomiting [12,13]. In the throes of physical and psychological dependency people want to evade drug withdrawals which can precipitate illegal drug seeking behaviour. Injecting is the preferred source of administration for street opioids but this carries health risks, including contracting and transmitting blood-borne diseases such as hepatitis B, hepatitis C, and HIV/Aids through sharing drug-injecting equipment. Hepatitis B and C are linked to liver diseases including cirrhosis and cancer, which can lead to death [14]. Worldwide it is estimated that 90% of new hepatitis C cases originate from unsafe drug injecting behaviour, while 80% of new HIV infections in eastern Europe and central Asia are associated with injecting drug use [4].

Considered a pharmacological and psychosocial intervention, OST addresses the chronic and relapsing progression of opioid use by preventing or mitigating harm through reducing illicit opiate use. Characterised as a harm reduction strategy to improve public health, OST provides opioid dependents with daily doses of oral methadone or buprenorphine (suboxone) tablets. Under the guidance of health professionals, opioid clients are assessed, treated, and monitored closely. Within Aotearoa/New Zealand opioids are classified as a Class A narcotic and governed by strict safety regulations. Takeaway doses of methadone (a dosage consumed without observation [11]) are considered a privilege and often occur after a client becomes “stable” and can comply with safety requirements. Although there are moral and ethical debates around the conditions that lead a person to OST, generally it is considered a successful and well-used evidence-based treatment modality that increases clients’ quality of life and overall health and wellbeing. While taking OST, clients avoid physical and mental intoxication and withdrawal symptoms. The aim is that they “stabilise” on a regular dose and possibly gain and maintain employment, rebuild family relationships, and improve physical health [11,15].

A small number of studies have explored OST in a disaster context, notably following the events of 9/11 and hurricanes in the USA. Findings emphasise a number of key issues in both client and health professionals’ experiences and access to medication following a disaster, including communication issues, access to records, adjustment of dosages, use of street drugs, withdrawal symptoms, and treatment services. These are discussed in more detail below.

Blance and colleagues [16] investigated the operations of 15 substance treatment programmes in New York City following 9/11. All seven methadone programmes remained open, with six having extended hours to help clients with transport problems, or “guests” who could not get to their methadone providers. However, clients were unable to phone to check if their treatment programmes were open as phone lines failed, while dosages could not be verified for people presenting at other providers than their own. Maxwell and colleagues [2] found similar issues with communication and access to dosage records following Hurricanes Katrina and Hurricane Rita (a month later) in Texas. For those residents of New Orleans who were evacuated, some clients received take-home opioid dosages before their evacuation. Many New Orleans health records were destroyed in the floods and staff could not be contacted with enquiries about dosages. When clients attended other clinics their methadone bottles were sometimes used to calculate dosages. Evacuees who had no identification and could not provide dosage levels were treated with “guest” doses for a few weeks, but eventually were placed on the local programme. When staff were unsure whether people claiming to be on an OST programme actually were, treatment was given only when these people demonstrated physical signs of withdrawals.

Following Hurricane Sandy in New York in 2012, McClure and colleagues [17] found that clinics were unable to operate and were relocated. There were problems with communication with regulatory agencies as well as verification of dosages and client status. The research explored two different OSTs and found stricter regulatory guidelines and procedures for methadone treatment compared to buprenorphine treatment, perhaps because buprenorphine is less likely to be abused and has less overdose risk [18]. Methadone programmes experienced more regulatory barriers, had more difficulty communicating with governing bodies, and an over-reliance on hospital emergency room dosing causing unsafe or suboptimal dosing levels. Although buprenorphine treatment had less regulatory barriers, there was no supporting coverage due to the limited number of providers. Tofighi et al. [19] investigated the effects of relocating a hospital centre that provided buprenorphine treatment after Hurricane Sandy. Over half of the 91 people they surveyed had treatment disruptions and coped by lowering doses themselves to make the medication last longer. When needed, additional buprenorphine was accessed through the relocated clinics, and also supplied by family members and drug dealers.

The system of treatment following a disaster event appears to be highly variable. Increased demand for methadone treatment for Katrina survivors meant that additional strain was placed on the system. Public and private funding streams contributed to this, with a low number of publicly funded programmes. However, most admissions occurred soon after the hurricane hit as people tried to regain access to a programme. Some emergency service staff without knowledge of opioid withdrawals thought that the diarrhoea experienced by opioid clients was symptomatic of a disease outbreak and tried to quarantine them [2]. Difficulties in accessing OST are also described by Bloodworth and colleagues [20] in their commentary on working in the Houston Astrodome following Hurricane Sandy. They describe how opioid dependents presented as soon as the medical clinics were opened in the Astrodome, asking for OST medication. However, due to drug enforcement regulations preventing narcotic prescriptions for dependency without official registration, methadone prescriptions were prohibited. Instead people were treated for withdrawal symptoms and within 48 h psychiatric services were referring and transporting people to local methadone centres. Controlled drugs (such as opioids) were not stocked at the on-site pharmacy because of security concerns and possible theft, so prescriptions were written and taken to local pharmacies.

Research has also highlighted how many OST clients experienced feelings of hopelessness, sadness, and desperation following 9/11, with the result that OST clinics reported illicit drug use by their clients as well as an increase in benzodiazepine use [16]. Pouget and colleagues [3] interviewed 300 people who inject drugs in order to explore their experiences after Hurricane Sandy. Consistent with research with health providers, these participants reported takeaway OST doses were problematic and when insufficient many used street drugs to avoid withdrawals. This highlights the importance of continuity of treatment for OST, as interruptions in dosing can affect the drug’s effectiveness and increase overdose risk for some.

The research conducted to date provides useful insight into what has occurred regarding OST within specific disaster contexts, however, we know little about how people address OST in the reduction and response phases of a disaster. The Aotearoa/New Zealand Ministry of Civil Defence and Emergency Management [21] define reduction as the process of analysing long-term risks and taking steps to minimise such risks, such as with preparedness planning, while response is the phase which involves actions immediately following an emergency that will help save lives and enable communities to recover. The current study sought to increase knowledge about OST and disaster planning within Aotearoa/New Zealand. Although it is difficult to accurately identify numbers of injecting drug users, it has been estimated that there were 5362 people on OST programmes as of June 2015 [22]. The Aotearoa/New Zealand Ministry of Health [11] views OST in terms of harm reduction, noting that “opioid dependence is a complex, relapsing condition requiring a model of treatment and care much like any other chronic health problem” (p. 1). In order to provide insight and knowledge that can inform the fields of disaster management and public health regarding OST, this study sought to explore the views and perceptions of service providers, health professionals, and emergency managers about the importance of OST provision and the needs of OST clients following a disaster.

## 2. Materials and Methods

### 2.1. Design

An in-depth qualitative interview-based study was employed to explore and gain insight into the views and perceptions of service workers, health professionals, and emergency managers regarding OST and disaster planning.

### 2.2. Procedure

Ethical approval was obtained by the institution’s Human Ethics Committee (Project identification code). Potential participants were identified as any health professional who had worked within or alongside OST, either at policy level or the coalface, and emergency managers with or without knowledge of OST. They were contacted either by “cold-call emails” or through snowballing techniques. Interviews were semi-structured and loosely covered the following topics: emergency management plans for OST dispensing, why OST is important to clients, and relevant issues that emergency management responders need to know. Interviews were audio-recorded and transcribed verbatim. Texts were organised for readability, redundant words such as “you know” and “um” were removed. Interviews ranged in length from 15 to 90 min. Data collection continued until data saturation was reached (a point at which no new information or themes emerge [23]).

### 2.3. Participants

Seventeen participants took part in individual interviews, including two emergency managers from District Health Boards, one community-focused emergency manager, two community-based pharmacists, one OST service pharmacist, four OST managers, one OST case worker, one OST administer/health and safety officer, one OST client advisor, one allied alcohol and drug professional, two Ministry of Health professionals working directly with OST services, and one Ministry of Health emergency manager. The sample represents a range of relevant health and emergency management professionals, covering the multidisciplinary nature and approach to OST. The research focused explicitly on those who have input into disaster planning and risk reduction and therefore did not include other professionals, such as disaster health care practitioners.

### 2.4. Analysis

A thematic analysis [24] was conducted to identify participants’ views and perceptions of OST in the reduction and immediate response phases of a disaster. The first step involved grouping data into 152 detailed codes, using NVivo 11 software. The codes were then discussed by the researchers and conceptually grouped into larger categories, which were considered in relation to each other and to the research aims. This led to further refinement and the identification of key themes and one central overarching theme.

## 3. Results

Three key themes were identified through the analysis, namely “health and wellbeing”, “developing an emergency management plan”, and “stock, dose verification, and scripts”. These were conceptualised as feeding into an overarching theme labelled “service continuity in OST preparedness planning”. Each theme is described in detail below.

### 3.1. Health and Wellbeing

Participants all communicated that clients’ physical health and wellbeing were central to OST continuity in a disaster context. Some participants expressed ethical care and concern for clients’ health as a reason to ensure treatment, consistent with the harm reduction philosophy underpinning OST in Aotearoa/New Zealand [11]. Participants noted how managing client health and safety in a disaster benefits everyone:

…on the whole clinicians all want the best for the client and the most effective way that benefits everybody, so I think you have to stand by them.

(Ministry of Health worker)

I think (the service) has a responsibility to minimise harm. That’s what the whole drug policy for us is about, that’s what our services are based on so if there is the potential for increased harm anywhere surely that’s a service role to look at how can we keep people safe?…so in a disaster situation yeah why wouldn’t you?

(OST staff)

“Minimising harm” is the responsibility of OST services, especially when there is risk of “increased harm” in a disaster context as expressed above. To reduce harm participants discussed how OST services should ensure opioid medication is dispensed to clients, especially those on daily medication:

…a lot of them dose daily, some are lucky enough to get some takeaways if they are in a stable environment maybe three times a week they’ll get takeaways.

(Pharmacist)

Here clients are viewed as “lucky” and “stable” if they have takeaway doses, implying that clients on daily doses are less “stable”. In this way, the dosage regime becomes an important consideration in a disaster context. Many participants noted medication as a key need for this group, as the following pharmacist points out:

We need to make sure that there’s continuity of their medication supply because if you do that at least psychologically they will be fine, and mentally they’ll be fine, if you don’t give them that then they’ll go into withdrawals and then you’ll get the complexity and the effects of psychological issues like depression, stress—all those kinds of things.

(Pharmacist)

OST staff reported that many clients have coexisting mental health disorders and trauma histories which will affect their ability to cope in a disaster context:

A lot of our clients, they’ve come from trauma backgrounds, which does impact on your capacity to be resilient in circumstances like this.

(OST staff)

Eight participants described how all clients (stable or not) go into “uncomfortable” physical withdrawals and psychological cravings without their daily dose. Participants emphasised the importance of getting the right opiate dose within a specific timeframe. Pharmacists pointed out that opiate withdrawals involve physical symptoms (cramps, sweating, and vomiting) which can start on the second day. Withdrawals are very uncomfortable, although not medically dangerous as some OST staff pointed out:

…nobody’s going to die without a dose, but you know that doesn’t help the distressed person who is extremely uncomfortable.

(OST staff)

The “distressing” nature of a disaster compounded with physical withdrawals makes the psychological implications of opiate withdrawals problematic in a disaster context, as demonstrated in the following pharmacists’ comments:

…the psychological thing of not picking up is probably a harder thing for them to deal with.

(Pharmacist)

…it becomes a bit of a physiological issue too that they can’t get into us or they can get in and see we’re not open and then they can’t get their way up to the (OST) clinic or they get there and there’s no-one there, it would be a pretty big problem.

(Pharmacist)

These issues were reinforced by an Alcohol and Drug (AoD) worker, who described opiate withdrawals in a disaster as a “harsh combination”. The participant went on to explain how “hanging out” (a slang term representing the physical and psychological effects of drug withdrawals) for medication could become dangerous:

…not physically able to get to a place of safety because you haven’t had your dose and you’re hanging out. So I do think it can become a matter of life and death or you know, maybe you can’t drive your car very well because you are hanging out, or maybe you have an accident.

(AoD worker)

The safety and wellbeing of the clients’ families, particularly dependents, was also mentioned by participants:

It’s crucial that there’s an alternative dispensing plan not only for the protection of the clients who are on OST but also for their dependents. So, for example, a disaster of some form has taken place and you have to get you and your family to a muster point or maybe you know you have to be physically able to go and gather water from a public water station, maybe you have to walk several k’s (kilometres) to be evacuated to a safe area and you’ve got dependent children and you’re hanging out.

(AoD worker)

Being able to access help or get to safety is essential. Participants also discussed clients with multiple health needs being on more than one medication. Although withdrawals from OST are not physically life threating in and of themselves, withdrawals for clients who are addicted to more than one substance could be physically dangerous if they are not clinically managed:

There’s often a lot of other stuff that people might be on, other meds as well, on benzodiazepines…if that gets suddenly cut off then there can be risks around seizures so there are some physical health concerns around the peripheral medications that quite a number of patients are on.

(OST staff)

Thus, access to other prescription drugs following a disaster is important for emergency management workers to consider. Many medications pose serious risks to health and wellbeing if abruptly discontinued. Overall participants’ talk around health and wellbeing centered on the importance of continuity of pharmacological services, which was also apparent in discussions around management planning for a disaster.

### 3.2. Developing an Emergency Management Plan

Many participants mentioned the 22 February 2011 Aotearoa/New Zealand Canterbury earthquake, which caused 185 people to lose their lives and many more to lose their homes. This event and its aftermath was described as inciting an urgent need for earthquake and disaster planning for OST services throughout Aotearoa/New Zealand, as shown in the following quote:

When Christchurch happened, that was the kind of smack in the face that, oh my God, you can’t not be prepared, you’ve got to have something in place and the manager was determined that we were going to have something in place.

(OST staff)

Participants described meeting with the Christchurch OST team, who shared their earthquake experiences and current emergency preparedness plan. Their descriptions highlighted collegial attitudes and practices, which often transpire after non-normal life events:

At the National Opioid Treatment providers meeting (Christchurch staff) talked about their experience and what that was like because you are trying to maintain a service, whilst at the same time dealing with personal stuff and there were people who were badly affected and they talked about having to move into an office space which suddenly had three other teams in it.

(OST staff)

OST staff also discussed the variability in OST planning. Some OST teams had District Health Board (DHB) emergency managers to assist with the development of preparedness plans, while others did not. The following quote describes attempts by one OST service to develop a plan:

…we had (Christchurch’s) disaster plan…so I adapted it…the pharmacist had some input and the doctors here had some input and then we sent it up to the emergency management people in the DHB and they went yeah okay we will look at it and we will consult with the various people and then we will get back to you. And time kept going on and on and eventually I went (banged table) it’s been a long time where is it and they came back with “it doesn’t meet the requirements for (this area) because of the way it is”.

(OST staff)

The time-consuming nature of preparedness planning is evident in this quote, even when there is assistance from colleagues and emergency managers. Participants described how the geographical terrain and physical location of OST services have to be factored into preparedness planning. For example, Wellington is located on an active geological fault line and the main arterial routes into the city could be cut off due to the valleys that lead into the city, whereas Auckland is more accessible. The risk for Auckland is volcanoes and tornadoes as well as infrastructure problems as discussed below. The unknown risk means planning strategies need to be broad according to a Ministry of Health participant, who notes that “all sorts of things can go down” and therefore OST services need to prepare for a variety of scenarios. Yet as participants commented, this makes planning challenging:

You can prepare and prepare and prepare but you don’t know until it happens and hopefully it doesn’t happen because it is all-hypothetical.

(OST staff)

…there’s a point where you think, you know, the plague, the plague, because people get so over wrought with the potentials of what could happen. They’re all about to walk outside and get attacked by a swarm of bees and collapse you know because the world is coming to an end (light laughter) so you can get totally caught up in it all.

(OST staff)

Here the various risk scenarios lea the participant to describe developing an OST emergency plan as an “over wrought” process, undermining it somewhat. There is little advice in the New Zealand Practice Guidelines for OST [11] on how to produce preparedness plans or deal with emergencies, which may leave sector workers feeling it is an impossible mission or feeling panicked:

That was part of the panic feel, you can’t dictate what could be a risk or what could be a disaster, you’ve just got to put in place what you can and deal with what you can.

(OST staff)

This quote also demonstrates how staff are pragmatic about planning, dealing with “what they can”. Some participants argued for a national strategy that could be adapted to use at a local level, while OST staff also discussed the importance of aligning with wider mental health and DHBs plans. However, there was a view that there is currently no global approach fitting the parts of planning together, as demonstrated in the following quote:

Well I don’t know that it’s well connected…the DHB plan whether it’s connected to the pharmacies’ plan, pharmacies have their own but whether ours fits with theirs, don’t know. The PHOs (Primary Health Organisations) don’t know how it all fits together. There’s no overall global kind of approach.

(OST staff)

A multidisciplinary system approach to OST requires multidisciplinary emergency planning. All OST teams consulted key stakeholders, such as community pharmacists and consumer representatives. Pharmacists play a significant role in OST provision because they dispense the medications and see OST clients most regularly, forming a relationship that is pivotal to treatment success. Some OST clinics have on-site pharmacies, however, once a client is stable dispensing shifts to a community pharmacist. Hence, a key concern for the OST teams and the emergency managers was communication with a dispensing pharmacist and primary health carers.

Some pharmacists outlined how they could draw from wider contacts, such as the Pharmacy Guild of New Zealand (an organisation that provides support to community pharmacies [25]) for guidance on emergency planning. The following quote from a community pharmacist highlights some of the current planning strategies:

Some of the pharmacists will have a plan, they know who their clientele are, they will have a list of clients that are on methadone, they will have stock if they are open…and they’ve got the scripts…If their business can still function they will look after their clients, with their stock, but…if their pharmacy’s damaged they cannot dispense at all then that’s where the issue is, we’d have to identify which client and send them to a pharmacy that is able.

(Pharmacist)

According to this pharmacist, if the community pharmacy can open they will “look after their clients”. This sense of professional responsibility was a common theme throughout the participants’ talks, demonstrating an awareness of the collective and multidisciplinary care approach that is required in OST. The discussion also highlighted the pivotal role of stock, scripting, and dosing issues as outlined in the next section.

### 3.3. Stock, Dose Verification, and Scripts

All participants, excluding the community emergency manager, highlighted three major concerns in a disaster context, namely access to OST stock, scripting problems, and dose verification. These were pertinent in discussions of a disaster that causes significant infrastructure damage and the inability of pharmacies to operate. Participants identified that an inability to access opioid medication has implications for service continuity and clients’ wellbeing.

According to some participants, extra stock is kept by some pharmacies; however, this is not made common knowledge because of safety issues. Despite this, stockpiling made sense as the following examples signify:

I know there are security reasons but it makes a lot more sense that pharmacists should have more of the stuff. Maybe not necessarily on site but there needs to be some designated place where there is a stockpile of these medications.

(OST staff)

I authorised that we can keep a much larger stock of methadone here and suboxone so we can actually manage for a few days.

(OST staff)

Having a “designated place”, even if “off site” was recommended as a way to deal with stock issues, while “managing” during the immediate response phase of a disaster was also an important emergency strategy. One of the pharmacists recommended that stockpiling be shared amongst allied services. Distributing stock through hospitals was also seen as a possible option that “made sense” to one OST staff member, while others had creative ideas for dispensing medication, such as area dispensing stations or a mobile dispensing van. However other OST staff were less sure about stockpiling due to pragmatic issues around timing and management:

You have a stockpile somewhere, but then how long do you keep the stockpile for? And then the store would have to go back to that, but who has access to that information about who’s on what?

(OST staff)

This excerpt also demonstrates concerns regarding dosing information. All participants discussed health records and dosing verification. According to the following Ministry of Health participant there is a national push towards health information technology (IT) more generally:

There is a huge investment in the health IT workspace so hopefully things like access to personal electronic health records. There is already the GP to GP (General Practitioner) record transfer; hopefully those “business as usual” processes are what we would be leveraging in a disaster that sees people moving out of their area.

(Ministry of Health worker)

The participant goes on to share how there are barriers that IT is trying to overcome, which include “suspicion” of technology:

There’s a lot of technological and organisational barriers that health IT have been working very hard to overcome. That goes down to people’s suspicion of having all of their medical information online and who can access it…outside of disaster management there is a huge investment in health IT.

(Ministry of Health worker)

“Investment in IT” is not specific to OST, but important nonetheless. Participants discussed electronic records specific to OST but connected to other health systems as a possible strategy for obtaining dosage information on clients:

Data on mental health clients, of which OST is part, is on a separate database so they were going away to have a chat to the IT people to see if it wouldn’t be possible to do the same thing, which at least means that somewhere in the Cloud you have got what the dose was in the last twenty-four hours.

(OST staff)

Ministry of Health has been driving very strongly to go to e-prescribing so that the data is held in the Cloud so it could be accessed at any site.

(Ministry of Health worker)

An e-prescribing cloud would enable data to be accessed at any site. However, while health IT was valued by some participants, others were concerned about computers failing in an emergency, and the consequences if all dosing information is stored on computers. All OST staff discussed physical dose registers as a way to overcome barriers to dose verification in these situations.

E-prescribing systems enable client records to be printed off, although this process is still trying to be worked through as the following participant recounted:

All the patients’ doses are entered…and the scripts are printed from that program, so what we’re trying to do now is the administrators here are trying to pull a list from that so that every week we can send a list to the emergency management people and it will say all the patients’ names, their NHI, and their dose.

(OST staff)

Concern was expressed about confidentiality of client information and staff in an emergency not necessarily having the time to check clients’ notes for dose amounts, and there were concerns about health privacy issues as shown below:

There are other privacy issues around how that information is stored and shared even though it’s really critical, but we have that list of where all these people live during an emergency—how do we share that in a way that’s safe outside of an emergency or including during an emergency…numerous challenges in this but it’s got to be looked at some time cos’ we can’t keep just ignoring it.

(Emergency Manger)

Our systems don’t talk to each other, it’s better now with electronic patient files, but unless they know to break glass and to have a look at somebody’s notes they don’t know that person’s on methadone, and who’s got the time in a busy department to do that?

(OST Staff)

A system breakdown is concerning especially in a disaster scenario when everyone is “busy”. OST staff discussed the possibility of clients carrying dosage information cards, although clients can lose cards and cards could be tampered with. One participant noted that card systems are used overseas, but she was unsure if they would work here because of privacy issues, recounting stories of clients being concerned about stigma and judgment from others if they are found carrying such cards. Another OST worker believed that clients will have information about dosing anyway:

When I see people clinically and they turn up for an assessment I say “oh what medication are you on” and they rummage around their bags and pull out whatever bottle, or a transcript from the pharmacy that says everything that they’re on, so most people (are) carrying around that sort of stuff, and so that would be proof of what you’re on really supposedly.

(OST staff)

Participants also commented that rigid dispensing practices might be more flexible in an emergency situation:

…in a disaster situation, probably they’re going to have to do things that the Ministry doesn’t want them to be doing because it’s a disaster.

(OST staff)

If a Pharmacist is dealing with someone he doesn’t know perhaps go to the local GP (to) get on the radio (and) call us and we try and verify who the patient is.

(Emergency Manager)

Participants highlighted the need to carefully manage the verifying of clients’ identities and dosing needs in a disaster. They also raised the importance of OST scripting and ability to supply clients:

How do we continue the supply to those patients? How do patients know where to go? If they usually go to their pharmacy and the pharmacy’s no longer available there needs to be some mechanism that patients can be notified.

(Ministry of Health worker)

Most participants mentioned communication as pivotal to any emergency plan. In the event of a major emergency the “worst case scenario” includes communication failures as outlined by the following emergency manager:

Worst case scenario I suppose is trying to get over things like communication failures and access problems and you know, we’ve got some limited capability with communications so we’ve got a radio telephone network in through the Health Sector across the Region.

(Emergency Manger)

Communication in OST involves scripting for a controlled drug, and participants rightly noted that there are only a limited number of specialists available in one region that can prescribe OST medication. Participants noted that if communication systems are not working, scripts cannot be signed off:

The regulatory environment for methadone adds complications as well because if you’re in specialist care and the specialist is unable to get where they need… how does that kind of work in terms of signing off scripts and that needs to be considered as well so it’s really complex.

(OST staff)

However, these prescriptions still require an authorised signature. One OST service kept scripts in a locked drawer and “the relevant people know where that key is”. While scripts could be produced, getting those scripts to appropriate pharmacies might still remain a challenge, and without scripts opioids cannot be legally dispensed:

Currently the OST scripts are all computer generated, assuming that there’s no power, (the manager) didn’t think there would be a problem to get the doctors to hand write the scripts. They haven’t quite figured out how they would get the scripts to the dispensing pharmacy which might be anywhere.

(OST staff)

Overall, the quotes in this theme highlight the technological and logistical challenges of dispensing alongside the regulatory boundaries of scripting. It also represents the participants’ views around taking a multidisciplinary approach to OST.

The three themes outlined here can be conceptualised as part of an overriding theme of ‘service continuity in OST preparedness planning’. While opioid medication is the key need for this group, service continuity was emphasised by participants as needing to be a priority during the response phase of a disaster. This was seen as essential for avoiding unnecessary physical and psychological distress for OST clients, their families, and wider communities. While overall findings held across all three professional groups of participants in this study, there were some differences determined by professional experience and knowledge. Alcohol and drug practitioners and OST health professionals hold greater knowledge about the rationale for continued service delivery and the characteristics of OST clients, while the emergency managers advocated for preparedness, continuity of care and minimisation of harm to OST clients, their families, and wider communities.

## 4. Discussion

Within emergency management, research into community disaster preparedness tends to apply mainstream population approaches or broad vulnerability perspectives overall (for example, see [26,27]) and investigates the everyday necessities of life (such as shelter, food, and medical care [5]) rather than the particular needs of vulnerable groups. While vulnerable populations are considered in preparedness planning, the needs of OST clients are not necessarily included within discussions of vulnerable population. The current findings demonstrated that for people working in OST, health services, and emergency management, opioid dependency presents a specific post-disaster condition that requires considered and empathetic understanding, planning, management, and attention. In the immediate response phase of a disaster, participants across three professional groups emphasised service continuity and ensuring accessibility and availability of opioid treatments. This was seen as important for the health and wellbeing of clients, developing suitable emergency plans, and in considering issues around stock, dose verification, and scripting in a disaster context. These findings are consistent with previous research highlighting issues that have occurred following disasters in the USA [2,16,17,19,20], particularly around getting access to medication, verifying correct dosages, and obtaining scripts.

Research [16] following the events of 9/11 and Hurricane Sandy [3] demonstrated that clients felt sad and feelings of despair after the disaster events, and when unable to access adequate OST they turned to street drugs to cope and avoid uncomfortable withdrawals. In this study, the health and wellbeing of OST clients was a significant concern for all participants. Providing a high standard of health practice is strongly advocated by the World Health Organisation [28] and aligns with Aotearoa/New Zealand’s commitment to the Sendai Framework for Disaster Risk Reduction [29]. This framework is founded on risk management strategies that promote the protection of all persons’ human rights, including the rights to health and safety following a disaster.

The central focus on service continuity in OST preparedness planning is also supported by previous research. For example, in their analysis of community emotional and psychosocial health following the Deepwater Horizon oil disaster in 2010, Yun, Lurie, and Hyde [30] argue that good disaster response requires awareness of any potential behavioural health outcomes, understanding the effected communities and any marginalised groups at risk, knowing the community support services, and utilising a multidisciplinary approach that includes local, state, and national coordination. They noted that disaster response should build on existing systems, by training mental health workers, using community engagement and peer support. These researchers also pointed out the need for better communication systems and updated disaster planning. Yun and colleagues [30] recommend that treatment programmes have disaster backup plans, with up-to-date data stored off site in a secure environment. However, as noted in the current study, if power is lost or OST services are closed due to building loss or damage, then paper copies of clients’ details and doses need to be available to ensure continuity of service (such as information on client dose, admission dates, takeaway regimes, and mental health status). Although some participants in the current study mentioned the possibility of clients having personal identification cards, previous research has found that the use of “smart” identification cards with photo and encrypted information of dosage and takeaway did not solve problems during the disaster because people lost their cards or the encrypted information could not be accessed [16]. Blance and colleagues [16] also assert that accommodating transient consumers requires support from hospital services. This could include hospitals dispensing methadone or the use of mobile vans to travel to clients. Such contingency plans were discussed by participants in the current study, who noted that collaboration between OST services is essential for this to be effective.

The specific context of OST is largely overlooked in disaster preparedness planning research [2], yet pre-existing mental and health conditions, including opioid-related disorders, produce poor health and wellbeing outcomes during and immediately following a disaster. These vulnerabilities are elevated when medication needs are not met. In her discussion on the post disaster response to people with disabilities following the Aotearoa/New Zealand Canterbury earthquake sequence, Mitchell [31] recognised that the failure of welfare centres to attend to the functional needs of people with disabilities was in part due to an absence of knowledge and understanding of disability and “disorder”. Training that values cultural specificity and multiplicity would prepare frontline responders, organisations, and emergency managers to deal with not only disability and wider mental health issues, but also the needs of people who inject drugs or are on OST.

Furthermore, while this research focused on health professionals and emergency managers’ views of OST provision following a disaster, it did not consider the perceptions of OST clients. These are crucial in any emergency planning around OST services, and future research is required to explore OST clients’ views and perspectives within this context. This will enable a more effective and holistic approach to disaster planning for OST.

## 5. Conclusions

In conclusion, people with opioid dependency present a specific group that needs to be considered in disaster planning and emergency management. The current findings demonstrate the importance of ensuring service continuity and the accessibility and availability of opioid treatments in a post-disaster context. While numerous lessons were learned from the Canterbury earthquakes in Aotearoa/New Zealand, it is recognised that all disaster events are specific and contextual. There is no prescribed textbook approach for the response and recovery phases of emergency management [32], but as the current participants noted, it is important to mitigate vulnerability and risks for OST clients. In this sense, preparedness planning that emphasises service continuity and values the unique and multiple needs of this population must be flexible and inclusive.
